# La-related protein 4 is enriched in vaccinia virus factories and is required for efficient viral replication in primary human fibroblasts

**DOI:** 10.1128/spectrum.01390-23

**Published:** 2023-08-18

**Authors:** Pragyesh Dhungel, Djamal Brahim Belhaouari, Zhilong Yang

**Affiliations:** 1 Division of Biology, Kansas State University, Manhattan, Kansas, USA; 2 Department of Veterinary Pathobiology, School of Veterinary Medicine & Biomedical Sciences, Texas A&M University, College Station, Texas, USA; 3 Department of Microbial Pathogenesis and Immunology, Texas A&M Health Science Center, Bryan, Texas, USA; Institute of Molecular Biology, Academia Sinica, Taipei, Taiwan

**Keywords:** LARP4, poxvirus, vaccinia virus, DNA replication, protein synthesis, viral factory, 5’-poly(A) leader

## Abstract

In addition to the 3′-poly(A) tail, vaccinia virus mRNAs synthesized after viral DNA replication (post-replicative mRNAs) possess a 5′-poly(A) leader that confers a translational advantage in virally infected cells. These mRNAs are synthesized in viral factories, the cytoplasmic compartment where vaccinia virus DNA replication, mRNA synthesis, and translation occur. However, a previous study indicates that the poly(A)-binding protein (PABPC1)—which has a well-established role in RNA stability and translation—is absent in the viral factories. This prompts the question of whether other poly(A)-binding proteins engage vaccinia virus post-replicative mRNA in viral factories. Here, in this study, we found that La-related protein 4 (LARP4), a poly(A) binding protein, was enriched in viral factories in multiple types of cells during vaccinia virus infection. Further studies showed that LARP4 enrichment in the viral factories required viral post-replicative gene expression and functional decapping enzymes encoded by vaccinia virus. We further showed that knockdown of LARP4 expression in human foreskin fibroblasts (HFFs) reduced vaccinia virus DNA replication, post-replicative protein levels, and viral production. Interestingly, the knockdown of LARP4 expression also reduced protein levels from transfected mRNA containing a 5′-poly(A) leader in vaccinia virus-infected and uninfected HFFs. Taken together, our results identified a poly(A)-binding protein, LARP4, being enriched in the vaccinia virus viral factories and facilitating viral replication in HFFs.

**IMPORTANCE:**

Vaccinia virus, the prototype poxvirus, encodes over 200 open reading frames (ORFs). Over 90 of vaccinia virus ORFs are transcribed post-viral DNA replication. All these mRNAs contain a 5′-poly(A) leader, as well as a 3′-poly(A) tail. They are synthesized in viral factories, where vaccinia virus DNA replication, mRNA synthesis, and translation occur. However, surprisingly, the poly(A) binding protein, PABPC1, that is important for mRNA metabolism and translation is not present in the viral factories, suggesting other poly(A) binding protein(s) may be present in viral factories. Here, we found another poly(A)-binding protein, La-related protein 4 (LARP4), enriched in viral factories during vaccinia virus infection. We also showed that LARP4 enrichment in the viral factories depends on viral post-replicative gene expression and functional viral decapping enzymes. The knockdown of LARP4 expression in human foreskin fibroblasts reduced vaccinia virus DNA replication, post-replicative gene expression, and viral production.

## INTRODUCTION

Poxviruses are a large family of double-stranded DNA viruses causing many significant diseases in humans and animals, including smallpox, one of the most notorious infectious diseases in human history (1). Mpox virus, the causative agent of mpox (previously named as monkeypox), is endemic in western and central Africa and, with an increasing presence in other regions of the world, poses a growing global threat to public health (2 – 4). Vaccinia virus (VACV) is the prototype poxvirus. It is a highly relevant surrogate to study high pathogenic poxviruses (e.g., mpox and smallpox viruses) due to their high similarity with >95% genome identity (5). VACV is also the vaccine against smallpox and mpox. VACV encodes over 200 open reading frames (ORFs) in its ~200 kbp genomes that are expressed in a cascade manner (6). While the early genes (118 ORFs) are expressed before viral DNA replication, the intermediate and late genes (91 ORFs) are expressed after viral DNA replication (7 – 9). The intermediate and late genes are collectively termed the post-replicative genes.

All the VACV post-replicative mRNAs have a 5′-poly(A) leader, in addition to a 3′-poly(A) tail, with heterogenous lengths that are likely generated by polymerase transcription slippage on a triple-thymine stretch of the template strand of the DNA promoter (10, 11). The role of the 3′-poly(A) tail in mRNA metabolism, including mRNA stability and translation, is well-established with countless publications. While it is still unclear whether the 5′-poly(A) leader regulates VACV post-replicative mRNA stability, studies from us and others indicate that the 5′-poly(A) confers a translational advantage in poxvirus-infected cells (12, 13). Our studies also indicate that the 5′-poly(A) is a cap-independent translation enhancer, which is promoted in VACV infection in a virally encoded decapping enzyme-dependent manner (14 – 16). Given that poxvirus post-replicative mRNAs have a 5′-poly(A) leader and 3′-poly(A) tail, it is conceivable that cellular poly(A)-binding proteins can be recruited to the viral factory by poxviruses for its replication. However, interestingly, the well-known poly(A)-binding protein, PABPC1, is located outside of VACV viral factories (17), where viral mRNAs are produced and translated (18), suggesting other cellular poly(A)-binding protein(s) may be involved.

RNA-binding proteins play diverse roles in regulating RNA metabolism. One such family of RNA-binding proteins is the La protein family, which comprises the La RNA-binding motifs and the RNA recognition motif (19). As a member of the La protein family, La-related protein 4 (LARP4) is known to bind to poly(A) RNA sequences (e.g., the 3′-poly(A) tail), the receptor for activated C kinase 1 (RACK1) and PABPC1 (20, 21). LARP4 promotes mRNA stability by binding to the 3′-poly(A) tail and preventing RNA deadenylation. The RNA-binding La motif, RNA recognition motif, and poly(A) binding protein-interacting domain in LARP4 are necessary for its mRNA stability enhancement function. Moreover, the predominantly cytoplasmic LARP4 is also postulated to play a role in translation as it is shown to associate with actively translating ribosomes, although the precise function is still unclear (19 – 21).

In the present study, with the objective to examine the roles of several poly(A)-binding proteins in VACV infection, we found that LARP4 was enriched in “virus factories,” a distinct cytoplasmic compartment where viral genome replication, intermediate and late transcription, and mRNA translation occur during VACV infection (18). The recruitment of LARP4 to viral factories requires VACV post-replicative gene expression and functional viral decapping enzymes. We further demonstrated that the knockdown of LARP4 was unfavorable for VACV replication and inhibited VACV replication at the DNA replication and post-DNA gene expression stages in primary human foreskin fibroblasts (HFFs). Taken together, our results identified the poly(A)-binding protein LARP4 as a pro-VACV replication host factor in primary human fibroblasts.

## RESULTS

### LARP4 is enriched in viral factories during VACV infection

**Fig 1:**
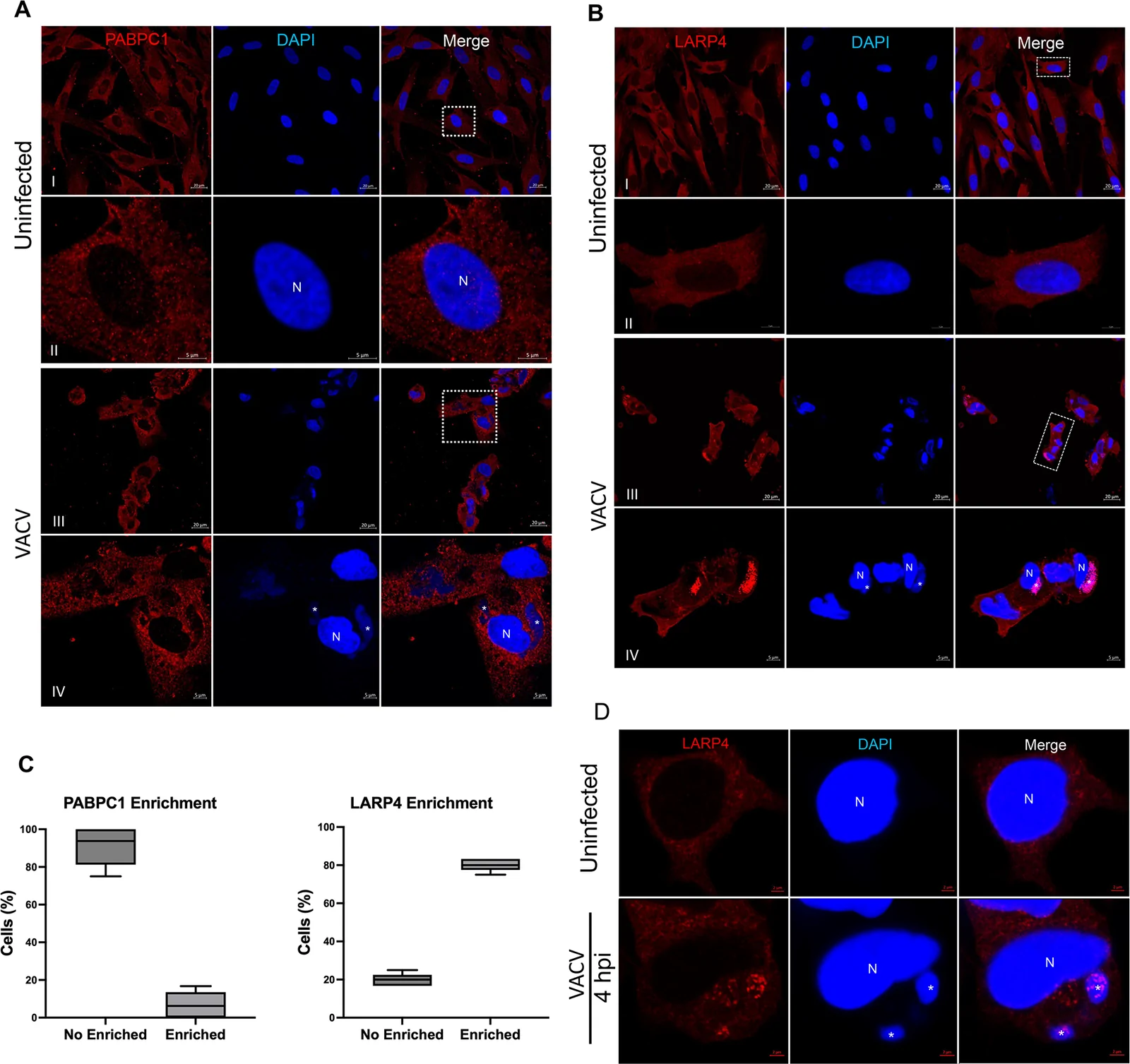
Enrichment of LARP4 in viral factories during VACV Infection of HFFs. (A) Confocal immunofluorescence microscopy of uninfected (I, II) and VACV-infected HFFs (III, IV) (MOI = 2 and 12 hpi). Anti-PABPC1 was used to determine the localization of PABPC1 (red), and DAPI (blue) was used to visualize cellular nuclei (N) and the viral factories (indicated by *). High-magnification images of the cell boxed in panel I and the infected boxed cells in panel III are shown in panels II and IV, respectively. The presented images are representative images of multiple fields of view. (B) Confocal immunofluorescence microscopy of uninfected (I, II) and VACV-infected HFFs (III, IV) (MOI = 2, 12 hpi). Anti-LARP4 was used to determine the localization of LARP4 (red), while DAPI staining was used to visualize cellular nuclei (N) and the viral factories (indicated by *). High-magnification images of the cell boxed in panel I and the infected boxed cells in panel III are shown in panels II and IV, respectively. The scale bars are 5 µm and 20 µm for high and low magnifications, respectively. (C) Quantification of the percentages of cells with PABPC1 and LARP4 enrichment in viral factories during VACV infection, a count of at least 25 cells was performed in different random confocal microscopy views. The resulting graph represents the percentage of cells with PABPC1 and LARP4 enrichment in viral factories. (D) Confocal immunofluorescence microscopy of uninfected and VACV-infected HFFs (MOI = 2). At 4 hpi, HFFs were fixed and stained with indicated antibodies or DAPI. Anti-LARP4 was used to determine the localization of LARP4 (red), and DAPI (blue) was used to stain cellular nuclei (N) and viral factories (indicated by *). The scale bars are 5 µm.

**Fig 2:**
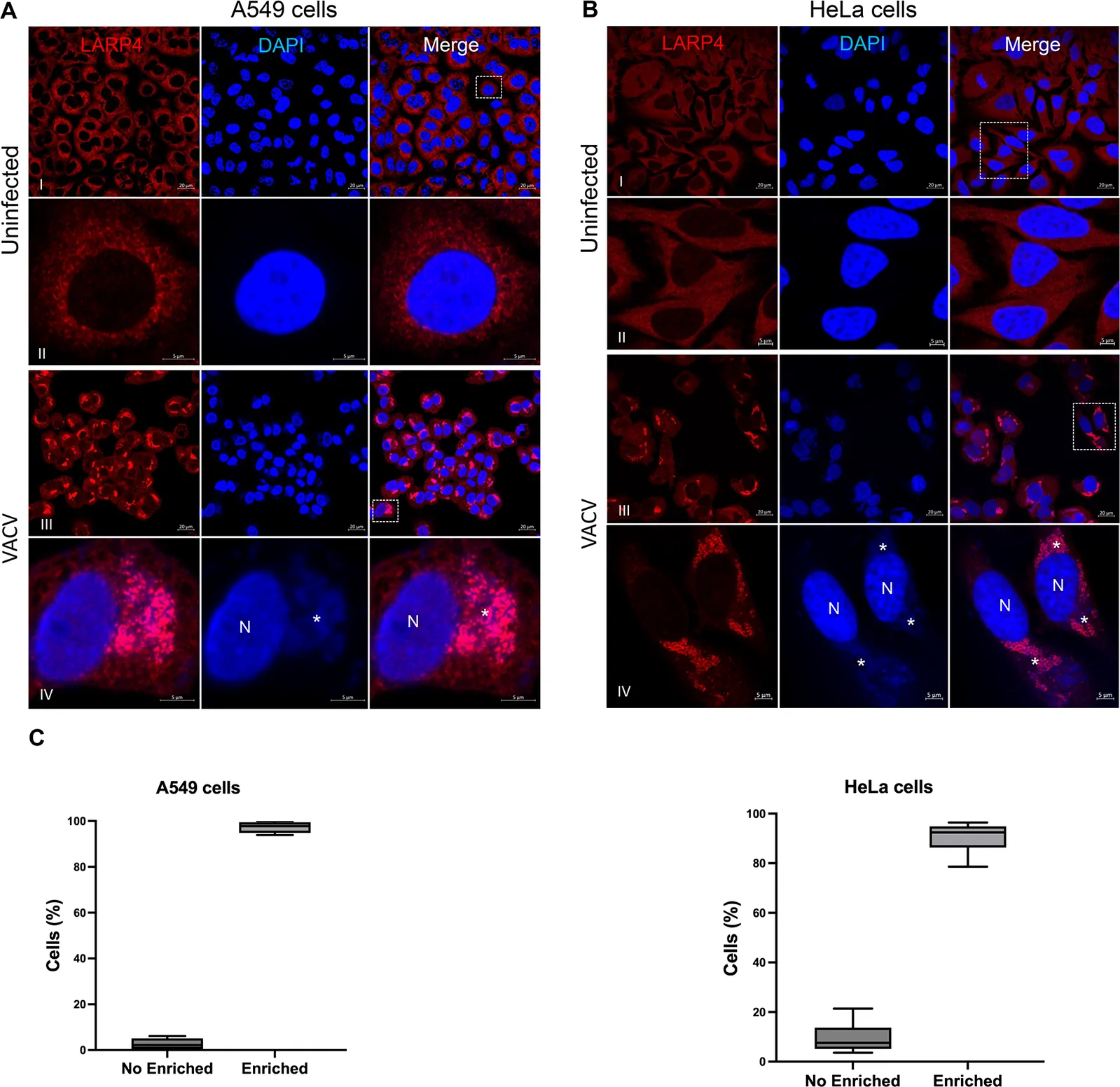
Enrichment of LARP4 in viral factories in VACV-infected A549 and HeLa cells. (A) Confocal immunofluorescence microscopy of uninfected (I, II) and VACV-infected A549 cells (III, IV) (MOI = 2 and 12 hpi). Anti-LARP4 was used to determine the localization of LARP4 (red), and DAPI (blue) was used to stain cellular nuclei (N) and viral factories (indicated by *). Panel II shows a high magnification of the cell boxed in panel I, and panel IV shows a high magnification of the infected boxed cells in panel III. (B) Confocal immunofluorescence microscopy of uninfected (I, II) and VACV-infected HeLa cells (III, IV) (MOI = 2 and 12 hpi). At 12 hpi, HeLa cells were fixed and stained with indicated antibodies. Anti-LARP4 was used to determine the localization of LARP4 (red), and DAPI (blue) was used to stain cellular nuclei (N) and viral factories (indicated by *). Panel II shows a high magnification of the cell boxed in panel I, and panel IV shows a high magnification of the infected boxed cells in panel III. In panels A and B, the presented images are the representatives of multiple fields of view. The scale bars are 5 µm and 20 µm for high and low magnifications, respectively. (C) The graphs show the quantification of cells with LARP4 enrichment in viral factories of VACV-infected A549 and HeLa cells. The percentage of cells with LARP4 enrichment in viral factories was counted across multiple random confocal microscopy views, with a total count of at least 160 cells per sample.

VACV replicates in the cytoplasm forming cytoplasmic DNA regions known as viral factories, where transcription and translation of viral intermediate and late mRNA occur (18). Using confocal immunofluorescence microscopy, we observed that a well-known poly(A)-binding protein, PABPC1, that plays a significant role in mRNA translation and stability by binding to poly(A) stretch of mRNA-, was not enriched in, but in fact, excluded from viral factories in VACV-infected HFFs (Fig. 1A and C). This result is consistent with a previous report that PABPC1 is distributed outside the viral factories in VACV-infected normal human dermal fibroblasts (17). Interestingly, another poly(A) binding protein, LARP4, was enriched in viral factories during VACV infection of HFFs, while it was dispersed in the cytoplasm of uninfected HFFs (Fig. 1B and C). The endogenous LARP4 enrichment in viral factories was detected as early as 4 h post-infection (hpi) (Fig. 1D). We also observed the enrichment of LARP4 in viral factories in VACV-infected A549 and HeLa cells (Fig. 2A through C). We did not observe a consistent pattern of increased LARP4 protein levels by Western blotting analysis during VACV infection (data not shown), suggesting the main effect of VACV infection is to re-localize and enrich LARP4 to viral factories.

### Enrichment of LARP4 in viral factories requires viral post-DNA replication events and functional VACV decapping enzymes

**Fig 3:**
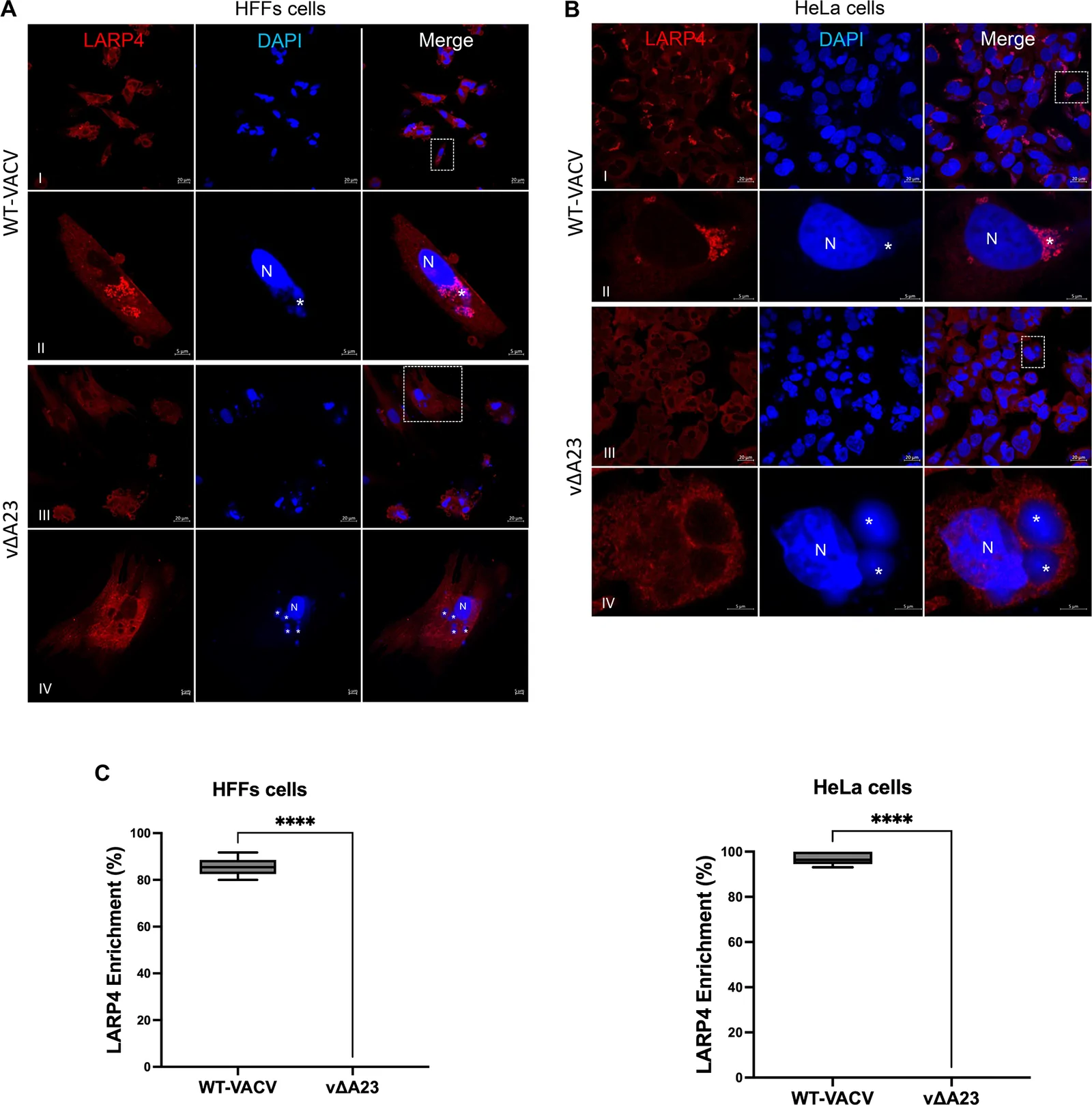
LARP4 enrichment in viral factories requires post-replicative gene expression. (A) Confocal immunofluorescence microscopy of WT-VACV-infected HFFs (I, II) and vΔA23 HFFs (III, IV) (MOI = 2 and 12 hpi). Anti-LARP4 was used to determine the localization of LARP4 (red), and DAPI (blue) was used to stain cellular nuclei (N) and viral factories (indicated by *). Panel II shows a high magnification of the cell boxed in panel I, and panel IV shows a high magnification of the cells boxed in panel III. (B) Confocal immunofluorescence microscopy of WT-VACV-infected HeLa cells (I, II) and vΔA23-infected HeLa cells (III, IV) (MOI = 2 and 12 hpi). Anti-LARP4 was used to determine the localization of LARP4 (red), and DAPI (blue) was used to stain cellular nuclei (N) and viral factories (indicated by *). Panel II shows a high magnification of the cell boxed in panel I, and panel IV shows a high magnification of the cells boxed in panel III. The presented images are the representatives of multiple fields of view. The scale bars are 5 µm and 20 µm for high and low magnifications, respectively. (C) Comparative analysis of LARP4 enrichment in viral factories of HFFs (left) and HeLa (right) infected with WT-VACV or vΔA23. *P* values were obtained using the Student’s *t* test, and the graph shows that LARP4 enrichment is significantly lower (*P* < 0.01) in cells infected with vΔA23 than in cells infected with WT-VACV.

VACV viral factories are formed after viral genomic DNA replication (18). To further investigate if VACV viral DNA replication alone without intermediate gene transcription and following events is sufficient for LARP4 enrichment, we utilized a recombinant VACV (vΔA23) with the ORF encoding intermediate transcription factor gene A23 deleted (22). While this virus undergoes genomic DNA replication and forms viral factories, the post-replicative gene expression is blocked at the step of intermediate transcription, and no intermediate and late mRNAs are produced (22). HFFs were infected with vΔA23, and subcellular localization of LARP4 was determined by confocal microscopy. At 12 hpi, we observed many viral factories. However, LARP4 was not enriched but excluded from the factories in HFFs and HeLa cells (Fig. 3A through C), indicating the requirement of post-DNA replicative event(s) to recruit LARP4 to viral factories.

**Fig 4:**
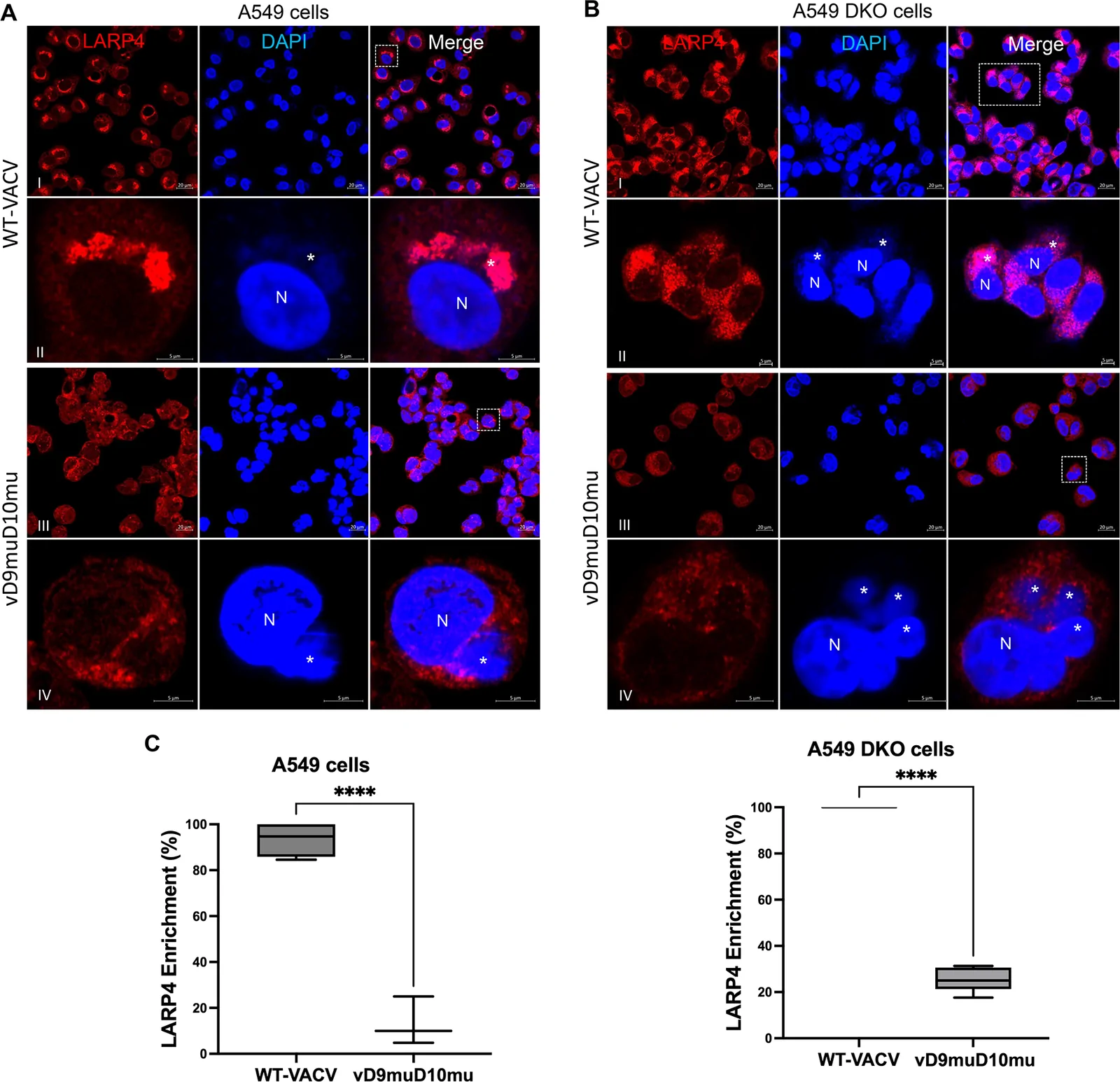
LARP4 Enrichment in viral factories requires functional VACV decapping enzymes. (A) Confocal immunofluorescence microscopy of WT-VACV-infected A549 cells (I, II) and vD9muD10mu-infected A549 cells (III, IV) (MOI = 2 and 12 hpi). Anti-LARP4 was used to determine the localization of LARP4 (red), and DAPI (blue) was used to stain cellular nuclei (N) and viral factories (indicated by *). Panel II shows a high magnification of the cell boxed in panel I, and panel IV shows a high magnification of the cells boxed in panel III. (B) Confocal immunofluorescence microscopy of WT-VACV-infected A549 DKO cells (I, II) and vD9muD10mu-infected A549 DKO cells (III, IV) (MOI = 2 and 12 hpi). Anti-LARP4 was used to determine the localization of LARP4 (red), and DAPI (blue) was used to stain cellular nuclei (N) and viral factories (indicated by *). Panel II shows a high magnification of the cell boxed in panel I, and panel IV shows a high magnification of the cells boxed in panel III. The presented images are the representatives of multiple fields of view. The scale bars are 5 µm and 20 µm for high and low magnifications, respectively. (C) Comparative analysis of LARP4 enrichment in viral factories in A549 (left) and A549 DKO (right) cells infected with WT-VACV or vD9muD10mu. The same confocal imaging specifications were used in the paired comparison. *P*-values were obtained using the Student’s *t* test, and the graph shows that LARP4 enrichment is significantly lower (*P* < 0.01) in cells infected with vD9muD10mu than in cells infected with WT-VACV.

Next, we used another recombinant VACV, vD9muD10mu, with both decapping enzymes (D9 and D10) inactivated by mutating their Nudix motifs that harbor their decapping enzyme activity. The mutations in D9 and D10 render them losing the ability to decap cellular and viral mRNAs to speed up RNA decay (23). VACV intermediate and late mRNAs are still efficiently produced in vD9muD10mu-infected cells, and the mRNAs are not decapped by viral D9/D10 (15, 16, 23, 24). To examine if LARP4 is enriched in viral factories during vD9muD10mu infection, we used the A549DKO cell line that allows VACV replication by knocking out two antiviral genes encoding PKR and RNase L. The knockout of RNase L also ensures intact cellular and viral mRNAs in vD9muD10mu infection, as VACV could activate RNase L by viral dsRNA for RNA cleavage. In A549DKO cells, viral intermediate and late mRNA are at higher levels than in WT-VACV infection, and proteins are also produced, though at lower levels than in WT-VACV-infected cells (24). Interestingly, our results revealed that LARP4 was minimally enriched in the viral factories of vD9muD10mu-infected A549 cells or A549DKO compared to WT-VACV-infected cells under the exact imaging specifications (Fig. 4A through C). These results suggest that functional decapping activities are necessary for LARP4 recruitment to the viral factories during VACV infection.

### Knockdown of LARP4 reduces VACV replication in HFFs

**Fig 5:**
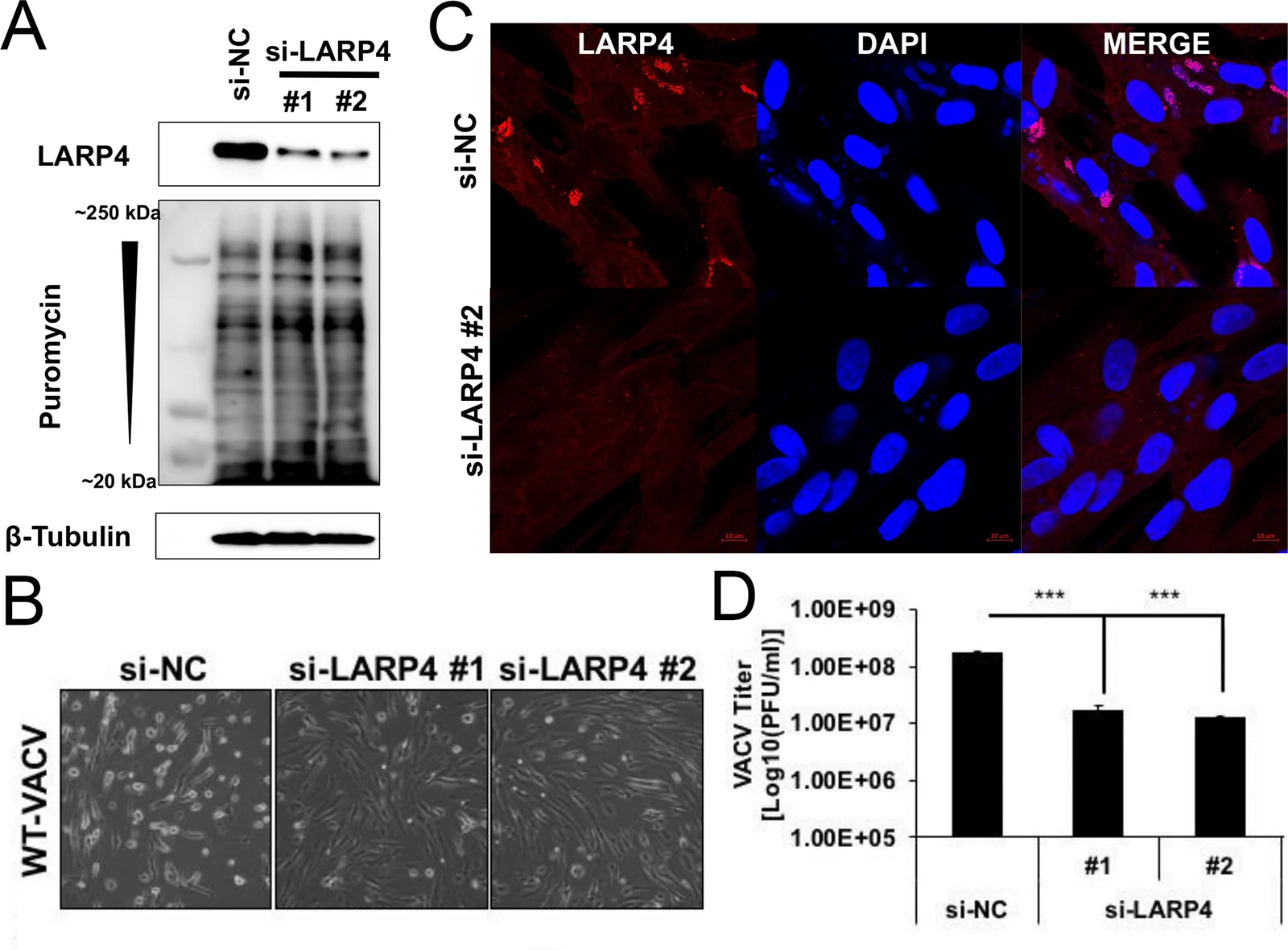
Knockdown of LARP4 protein expression reduces VACV replication. (A) HFFs were transfected with negative control siRNA (si-NC) or siRNAs targeting LARP4. Nascent protein synthesis was determined by treating cells and labeling nascent protein with puromycin (10 µg/mL) for 20 min at 37°C. Levels of LARP4, puromycin-labeled nascent protein, and β-tubulin proteins were detected using specific antibodies. (B) At 72 h post-siRNA transfection, HFFs were infected with VACV at an MOI of 3. VACV-infected cells were observed 12 h post-infection by microscopy. (C) LARP4 staining using anti-LARP4 antibody in si-LARP4-treated cells. Confocal immunofluorescence microscopy of HFFs treated with si-LARP4 (#2) or siNC and infected with WT-VACV. Anti-LARP4 (red) and DAPI (blue) were used to stain LARP4 protein, cellular nuclei, and/or viral factories, respectively. (D) At 24 hpi, HFFs were collected for plaque assay. The titers of the VACV during indicated treatment were determined by plaque assay using BSC-1 cells. Error bars represent the standard deviation (SD) of at least three experiments. *P* values were obtained using the Student’s *t* test; ***P value <0.001.

In order to investigate the role of LARP4 in VACV replication, we knocked down LARP4 expression in HFFs using two siRNAs targeting different regions of LARP4 mRNA. Successful knockdown of LARP4 protein levels was confirmed by Western blot analysis (Fig. 5A). The siRNA-mediated depletion of LARP4 in HFFs did not exert a noticeable decrease in nascent cellular protein synthesis using puromycin labeling (Fig. 5A), suggesting an indiscernible effect on overall cellular protein synthesis rate in HFFs. To assess VACV replication in LARP4-depleted HFFs, the cells were infected with VACV at a multiplicity of infection (MOI) of 3. Interestingly, at 12 h post-VACV infection, the HFFs with LARP4 siRNA exhibited a less severe cytopathic effect than the control siRNA-treated HFFs (Fig. 5B), suggesting that the LARP4 siRNA treatment may limit VACV replication. Anti-LARP4 staining in siRNA-treated, VACV-infected HFFs indicated that LARP4 was not enriched and the signal was weaker than in non-specific siRNA-treated cells (Fig. 5C). The result also indicated the specificity of the antibody in recognizing LARP4. We determined VACV titers 24 hpi by a plaque assay. Compared to the control siRNAs, LARP4 siRNAs (#1 and #2) reduced VACV titers by 10-fold and 14-fold, respectively (Fig. 5D). Taken together, these results indicate that LARP4 facilitates optimal VACV replication in HFFs.

### Knockdown of LARP4 has little effect on VACV early gene expression but reduces viral DNA and post-replicative protein levels

**Fig 6:**
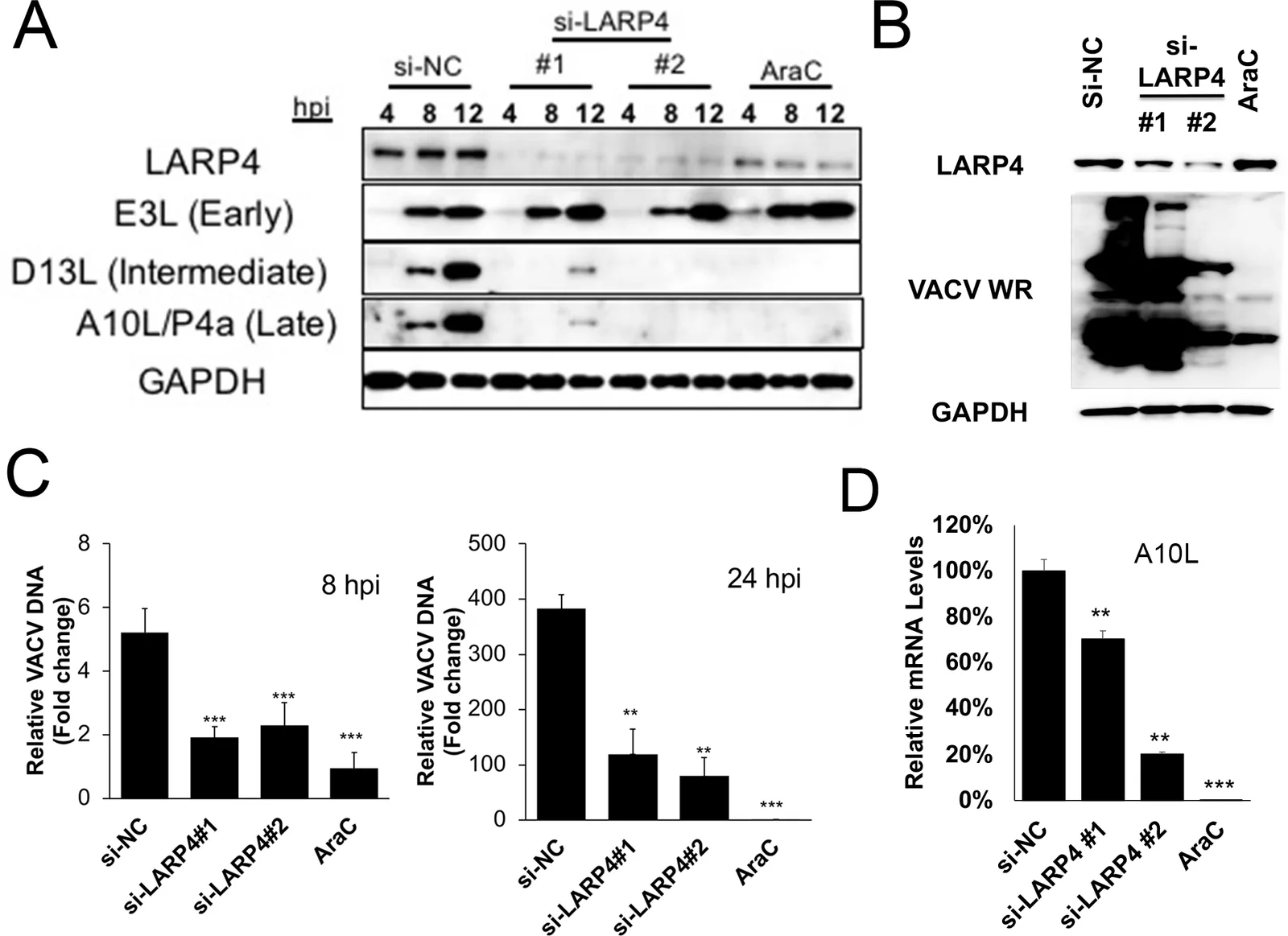
Depletion of LARP4 reduces post-replicative gene expression and viral DNA replication. (A) HFFs cells were transfected with negative control siRNA (si-NC) or LARP4 siRNAs. Seventy-two hours post-transfection, HFFs were infected with VACV at an MOI of 5. AraC (40 µg/mL) treatment was used as a positive control. The HFFs were lysed, and the lysates were analyzed by Western blotting analysis to determine the levels of LARP4 at different time points (4, 8, and 12 hpi). (B) HFFs cells were transfected with negative control siRNA (si-NC) or LARP4 siRNAs. Seventy-two hours post-transfection, HFFs were infected with VACV at an MOI of 5. The HFFs were lysed, and the lysates were analyzed by Western blotting analysis using the anti-VACV serum at 24 hpi. (C) At 1, 8, or 24 hpi, total DNA was extracted from the infected HFFs with indicated treatment. Levels of VACV DNA were determined and represented as fold changes of VACV DNA at 8 or 24 hpi to 1 hpi. (D) At 12 hpi, relative levels of VACV late gene A10L mRNA were measured by qRT-PCR. 18S rRNA was used for normalization. Error bars represent the standard deviation (SD) of at least three experiments. *P* values were obtained using Student’s *t* test; *P value <0.05, **P value <0.01, ***P value <0.001.

VACV replication is divided into entry, early gene expression, viral DNA replication, intermediate and late gene expression, virion assembly, and release. We utilized Western blotting analysis to investigate the impact of LARP4 siRNA treatment on viral protein levels in HFFs. Our results showed a significant decrease in viral post-replicative protein levels in LARP4 siRNA-treated cells, indicating that LARP4 is needed for efficient viral post-replicative protein production. As shown in Fig. 6A, HFFs infected with VACV at different time points (4, 8, and 12 hpi) revealed that LARP4 siRNA treatment did not affect viral early protein E3 levels but led to a substantial reduction in the synthesis of VACV intermediate protein D13 and late protein A10. A Western blotting analysis using anti-VACV serum indicated less viral protein expression in LARP4 siRNA-treated cells (Fig. 6B). Additionally, we evaluated VACV DNA replication by quantitative PCR using DNA extracted from control and LARP4 siRNA transfected VACV-infected HFFs. AraC, an inhibitor of DNA replication, was used as a positive control. The results showed a moderate yet significant reduction (two- to fivefold) in VACV DNA levels after LARP4 knockdown (Fig. 6C). As expected, AraC treatment (positive control) significantly reduced VACV DNA levels (Fig. 6C). The reduction of mRNA levels of a late gene (A10L) in HFFs with LARP4 knockdown was observed (Fig. 6D). These findings suggest that LARP4 starts to impact VACV replication at the genome replication stage and reduces viral post-replicative gene expression.

### Knockdown of LARP4 in HFFs decreases protein production from transfected mRNA with a 5′-poly(A) leader

**Fig 7:**
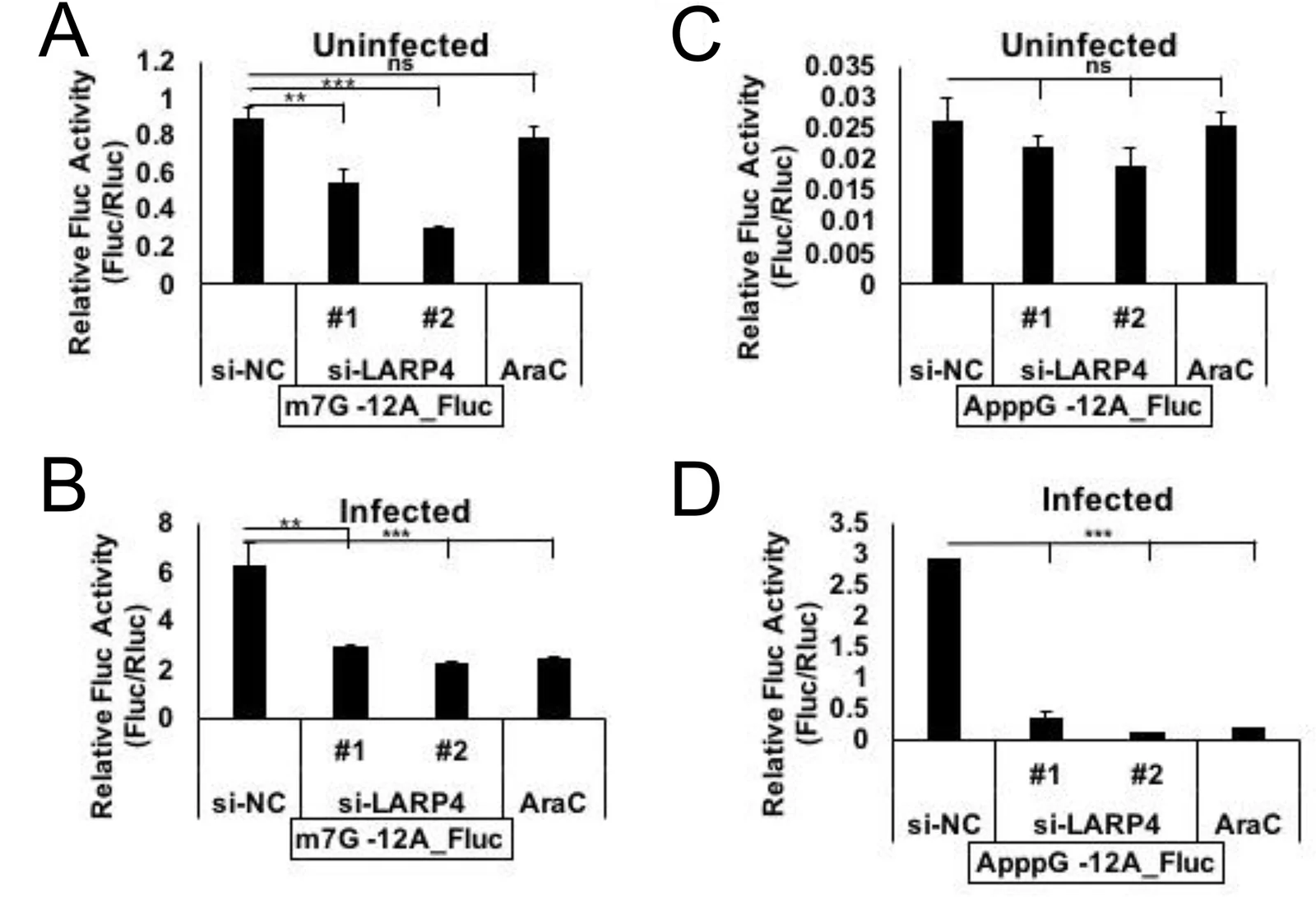
LARP4 is required for protein synthesis from tranfected mRNA with a 5′-poly(A) leader in HFFs. HFFs were either infected with VACV (MOI = 5) (B and D) or kept uninfected (A and C) after 72 h post-transfection of control or LARP4 siRNAs. AraC (40 µg/mL) treatment was initiated after 1 h of infection or uninfected condition. 5′-Poly(A) leader containing Fluc reporter mRNA and Kozak-headed Rluc mRNA were transfected into uninfected and VACV-infected HFFs at 12 hpi. Fluc and Rluc mRNAs were capped by either m^7^G (A and B) or ApppG (C and D). Relative luciferase activities were measured 5 h after reporter mRNA transfection by normalizing 5′-Poly(A) leader-driven Fluc activity by co-transfected Kozak sequence-driven Rluc activity. Error bars represent the standard deviation (SD) of at least three experiments. *P* values were obtained using the Student’s *t* test; **P value <0.01, ***P value <0.001, ns = not significant (i.e., *P* value > 0.05).

All VACV post-replicative mRNAs have a 5′-poly(A) leader that confers a translational advantage in VACV-infected cells (14). LARP4 is a poly(A)-binding protein (20). We asked if the knockdown of LARP4 affected protein synthesis from mRNA with a 5′-poly(A) leader. Following LARP4 depletion by two siRNAs, respectively, HFFs were mock-infected or infected with VACV (MOI = 5). The 5′-poly(A) leader-containing the Fluc reporter mRNA and Kozak-headed Rluc mRNA were co-transfected into uninfected and VACV-infected HFFs at 12 hpi, respectively, as described previously (14, 25). The co-transfection of RNAs with a 5′-poly(A) and a non-5′-poly(A) UTR could indicate differential effects of LARP4 knockdown on proteins synthesized from these mRNAs. Fluc and Rluc reporter mRNAs were either m^7^G capped (Fig. 7A and B) or ApppG capped (Fig. 7C and D). The translation from ApppG-capped RNA indicates cap-independent translation as this cap analog does not initiate cap-dependent translation initiation assembly (26, 27). Relative luciferase activities of 5′-poly(A) leader-driven Fluc activities were normalized by co-transfected Kozak sequence-driven Rluc activities. LARP4 knockdown decreased the normalized luciferase activities from the mRNA with an m^7^G capped 5′-poly(A) leader in uninfected HFFs (Fig. 7A) and VACV-infected HFFs (Fig. 7B). Similarly, LARP4 depletion also significantly decreased the normalized luciferase activities from mRNA with an ApppG-capped 5′-poly(A) leader mRNA in VACV-infected cells (Fig. 7D). No significant change in luciferase activities from the ApppG-capped 5′-poly(A) leader mRNA was observed in uninfected cells (Fig. 7C), which had very low Fluc expression as expected. Our previous study indicated that 5′-poly(A) leader-mediated translation advantage in VACV-infected cells occurs during the post-replicative stage of VACV gene expression (14). The post-replicative gene expression does not happen in AraC-treated cells since the AraC blocks VACV genome replication. Unlike in VACV-infected cells, AraC treatment did not alter protein production from mRNA with a 5′-poly(A) leader in uninfected HFFs (Fig. 7A). These results suggest a possible role of LARP4 in 5′-poly(A) leader-mediated translation in both uninfected and VACV-infected cells, including cap-independent translation enhancement in VACV-infected HFFs.

## DISCUSSION

While it has long been suspected that one or more poly(A)-binding proteins are required explicitly for VACV replication, it is intriguing that the PABPC1 is excluded from the viral factories (17). The localization of PABPC1 outside of the viral factories suggests a non-essential role of this protein in poxvirus mRNA metabolism and translation in viral factories, as viral mRNAs are produced and can be translated in the factories (17, 18). Our data that LARP4, a poly(A)-binding protein, translocated to viral factories during VACV replication suggests the virus utilizes this specific poly(A)-binding protein to facilitate viral replication. One previous study showed that LARP4-NTD (N-terminal domain) is essential for poly(A) RNA binding (28). LARP4-NTD also contains the PAM2w motif, which is necessary for interaction with the MLLE domains of PABPC1. Cruz-Gallardo et al. demonstrated that LARP4-NTD binding is mutually exclusive with a PABP or poly(A) RNA sequence (28). This provides a reasonable explanation for the observation that PABPC1 is excluded from the viral factories, while LARP4 is enriched in the viral factories.

Intriguingly, the enrichment of LARP4 in VACV viral factories could not occur in vΔA23- and vD9muD10mu-infected cells. In vΔA23-infected cells, while viral early mRNAs, proteins are produced, and viral genomic DNA is replicated at high levels, viral intermediate mRNAs are not produced due to the lack of one of the intermediate transcription factors A23 (22). Subsequently, the following steps of VACV replication, including late mRNA synthesis, are also blocked. As LARP4 is a poly(A)-binding protein, this finding suggests an intermediate and late viral mRNA synthesis requirement in the factories to recruit LARP4. However, the data that LARP4 was not enriched in viral factories in vD9muD10mu-infected cells suggests the intermediate and late gene expression is not sufficient to recruit LARP4 to viral factories, as previous studies indicated that viral post-replicative gene expression, including RNA and protein synthesis, occurred in vD9muD10mu-infected cells, especially in A549DKO cells (16, 23, 24). One possible explanation for this finding is that the inactivation of the two decapping enzymes, D9 and D10, delayed and reduced the degradation of cellular mRNAs that may retain LARP4 outside the viral factories. As D10 is an intermediate protein (8) that is not expressed in vΔA23-infected cells, it is possible that the failure to promptly degrade host cell mRNAs in vΔA23 infection prevents the recruitment of LARP4 to viral factories.

Further investigations are needed to elucidate how LARP4 regulates VACV replication. LARP4 possibly affects VACV replication through multiple mechanisms, including regulating viral DNA replication, mRNA stability, and translation efficiency, as well as other indirect mechanisms. It is intriguing that knockdown of LARP4 reduced VACV DNA replication (Fig. 6C), as there is no previous report for a role of LARP4 in DNA replication. The effect could be through an indirect mechanism. As LARP4 is a poly(A) binding protein with well-established role in promoting mRNA stability (20), it may bind VACV mRNA through the 5′-poly(A) leader and/or the 3′-poly(A) tail in the viral factories to enhance viral mRNA stability in addition to affecting RNA translation. We have previously shown that VACV decapping enzyme D10 co-localizes with mitochondria, providing a spatial mechanism for the decapping enzyme to preferentially induce the degradation of cellular mRNAs over viral mRNAs (16). The enhancement of viral mRNA stability by LARP4 may provide another mechanism for viral mRNAs to be less likely degraded. Our results also suggest that LARP4 may facilitate the translation of mRNAs with a 5′-poly(A) leader, including cap-independent translation, during VACV infection. This possibility is suggested by our results using an RNA-based reporter system in Fig. 7, with the caveat that the transfected RNA stability may also be reduced in LARP4 knockdown cells. Additionally, VACV decapping enzymes are required to efficiently translate RNAs with a 5′-poly(A) leader (15), which is consistent with the finding in this study that LARP4 is not enriched in the viral factories in vD9muD10mu-infected cells. Additionally, LARP4 has been known to positively regulate the general translation of mRNAs that do not have a 5′ poly(A) leader (20).

Furthermore, LARP4 may indirectly regulate VACV DNA replication, mRNA stability, and translation. Such a possibility is suggested by reduced VACV genomic DNA replication in LARP4 knockdown cells and its cell type-specific role. Knockdown of LARP4 significantly reduced VACV replication in primary HFFs. However, we did not observe a similar effect on VACV replication in HeLa cells with LARP4 knocked down (data not shown). These observations indicate a cell type-specific effect of LARP4 on VACV replication, which may attribute to differential expression of LARP4 and its isoforms or other poly(A)-binding proteins in different cell types due to their transformation statuses or tissue origins. One such indirect role that LARP4 may play in VACV replication is through LARP4 regulation of the expression and stability of mRNAs encoding for interferon (IFN) and antiviral proteins, which is suggested by LARP4’s differential roles in different classes of RNAs (21, 29). In transformed cells (e.g., HeLa cells), it is not uncommon to have impaired interferon response and to lack the ability to activate some types of antiviral responses (30, 31). LARP4 depletion in primary HFFs may promote IFN and other antiviral pathways during VACV infection, which may in turn reduce viral DNA replication, as well as viral mRNA transcription and translation indirectly.

Taken together, our data presented in this study demonstrated that LARP4 is enriched in viral factories during VACV replication. The protein is required for efficient VACV replication in primary HFFs. The mechanism by which LARP4 influences VACV replication is of great interest to pursue in future studies.

## MATERIALS AND METHODS

### Cell culture and virus infection

HFFs (a gift from Dr. Nicholas Wallace) were cultured in Dulbecco’s modified Eagle’s medium (Quality Biological) containing 10% fetal bovine serum (Peak Serum) and 2 mM L-glutamine (Quality Biological). Cells were incubated in a 5% CO_2_ atmosphere at 37°C. VACV Western Reserve strain (ATCC VR-1354), vD9muD10mu with both decapping enzymes inactivated, and vΔA23 with intermediate transcription factor gene A23 deleted were kindly provided by Dr. Bernard Moss and were described previously (22, 23). Virus titer was determined by plaque assay as described elsewhere (32).

### Antibodies and chemical inhibitors

Anti-LARP4 (A5108) and anti-PABPC1 (A14872) antibodies were purchased from ABclonal Inc. Anti-GAPDH (sc-365062 HRP) antibodies were purchased from Santa Cruz Biotechnology. Anti-puromycin (MABE343) was purchased from Sigma-Aldrich. Anti-D13L and anti-A10L/P4a antibodies were gifts from Dr. Bernard Moss. The anti-E3L antibody was present from Dr. Yan Xiang. Cytosine arabinoside (AraC) was purchased from Sigma-Aldrich.

### Western blotting analysis and nascent protein analysis

Protein levels were evaluated by preparing samples as described previously (33). The sample was resolved in the SDS-PAGE gel and transferred to a polyvinylidene difluoride membrane. For detection of protein, membranes were blocked in either 5% bovine serum albumin (BSA) or 3% milk for 1 h at room temperature, followed by incubation with primary antibody for 1 h at room temperature or overnight at 4°C and finally, incubation with secondary antibody added in 1× TBST (with either 3% milk or 5% BSA).

Nascent protein synthesis was determined by treating the cells with puromycin (10 µg/mL, P8833, Sigma-Aldrich) for 20 min at 37°C (16). Treatment was aborted by removing the cell culture media containing puromycin and washing once with 1× phosphate-buffered saline (PBS). NP-40 lysis buffer was added directly to the cells, which were subsequently scrapped. Lysis was carried out by rotating the sample at 4°C for 30 min and centrifuging at 12,000 × *g* at 4°C for 10 min. The supernatant was used for sample preparation and evaluated by western blot analysis.

### *In vitro* transcribed RNA-based luciferase assay

The RNA-based luciferase assay was used to determine the translation of 5′-poly(A) leader containing mRNA during LARP4 depletion using the protocol described previously (14, 25). The RNA used in this study was capped with either m^7^G (Anti-Reverse Cap Analog, S1411L) or ApppG cap analog (S1406S, New England Biolabs).

### Knockdown using small interfering RNA

The small interfering RNAs (siRNAs) used for this study were purchased from Integrated DNA Technologies. Lipofectamine RNAiMax (Thermo Fisher Scientific) was used to transfect siLARP4 or siNC (Negative Control) to HFFs. The siRNAs were used at the final concentration of 12.5 nM. At 72 h post-transfection, the cells were either collected for Western blotting analysis to determine siRNA-mediated depletion of the target protein or infected with VACV for further analysis.

### Confocal immunofluorescence assay

After desired treatment, the cells were fixed with 4% formaldehyde (28908, Thermo Fisher Scientific) in 1× PBS for 20 min. The cells were then permeabilized with 0.1% Triton X-100 (9002-93-1, Thermo Fisher Scientific) in 1× PBS for 15 min. The cells were washed 3× with 1× PBS and blocked for 1 h at room temperature with 2% BSA containing 1× PBS. Then, the cells were incubated with the desired primary antibody (1:100 dilution in 2% BSA containing 1× PBS) for 1 h at room temperature. Again, after 3× washes, the cells were incubated with a secondary antibody conjugated with a fluorophore (1:500 dilution in 2% BSA containing 1× PBS) for 1 h at room temperature. Following 2× washes with 0.1% Triton X-100 in 1× PBS, DAPI stain (5 µM) in 1× PBS was added to the cells for 20 min at room temperature. Finally, 2× washes with 1× PBS were done to remove any unbound DAPI stain. The image was taken using Carl Zeiss LSM 880 confocal microscope. ZEISS Efficient Navigation software was used to analyze the images.

### Quantitative real-time PCR

For DNA, total DNA was extracted from mock- or VACV-infected cells using E.Z.N.A. Blood DNA Kit. For RNA, RNA was extracted using a TRIzol reagent (Ambion) and purified using a PureLink RNA Mini Kit (Thermo Fisher Scientific). The purified RNA was used to synthesize cDNA using SuperScript III First-strand synthesis (Invitrogen) with the addition of either random hexamer or oligo-dT primers. Relative viral DNA (or cDNA) levels were quantified by CFX96 real-time PCR instrument (Bio-Rad) with an All-in-one 2× qPCR mix (GeneCopoeia) and primers specific for indicated genes or VACV genome, respectively. GAPDH or 18S rRNA was used as the internal control.
